# Context-dependent rewiring of dual-function proteins in cancer: a sequential strategy to restore apoptosis

**DOI:** 10.3389/fonc.2025.1675537

**Published:** 2025-10-07

**Authors:** Leon Strzadala

**Affiliations:** Hirszfeld Institute of Immunology and Experimental Therapy, Polish Academy of Sciences, Wroclaw, Poland

**Keywords:** apoptosis, tumor microenvironment, p53, Ras, HIF-1α, BNIP3, NF-κB, sequencing

## Abstract

Resistance to programmed cell death is a defining hallmark of cancer and a persistent barrier to successful therapy. Dual-function proteins such as p53, Ras, HIF-1α, BNIP3, and NF-κB act as molecular switches that determine cell fate between apoptosis and survival. In tumors, these proteins are deregulated not only by intrinsic mutations but also by extrinsic signals from the tumor microenvironment (TME). This Mini Review critically analyzes previous therapeutic approaches, emphasizing overlooked mechanisms such as Ras-mediated suppression of p53. It proposes a sequential therapeutic strategy: first, dismantling TME adaptations (hypoxia, inflammation, protective autophagy); second, inhibiting oncogenic Ras signaling; and third, restoring p53 activity. The phased approach integrates biomarker-guided patient stratification, recognizes tumor–microenvironment co-evolution, and highlights how resistance evolves over time. Although the concept does not resolve all challenges, it outlines a rational framework for restoring apoptotic competence and provides a pathway for translational and clinical testing.

## 1 Introduction

Avoidance of programmed cell death is a hallmark of cancer and a fundamental challenge in oncology (1). This capability allows malignant cells to persist despite genomic instability, oncogenic signaling, and cytotoxic treatments. Crucially, apoptotic resistance emerges not only from cell-intrinsic genetic alterations but also from tumor microenvironment (TME) cues that collectively buffer pro-death signals (2). Hypoxia stabilizes hypoxia-inducible factor 1α (HIF-1α), promoting glycolysis, angiogenesis, and survival under metabolic stress (3). Inflammatory cytokines produced by stromal and immune cells chronically activate NF-κB, reinforcing survival, angiogenesis, and immune evasion (4). BNIP3, a BH3-only protein with pro-apoptotic potential, is frequently rewired to promote protective mitophagy and macroautophagy in stressed tumor cells (5). Finally, oncogenic Ras programs resistance by repressing p53 through multiple layers of signaling crosstalk, thereby raising the threshold for apoptosis (6). These mechanisms do not operate in isolation; rather, they co-evolve as transformed cells interact with and remodel their microenvironment, generating resilient ecological niches that adapt under therapeutic pressure (7). Addressing apoptosis resistance therefore requires a systems-level approach that integrates both intracellular wiring and microenvironmental context.

## 2 Dual-function proteins as molecular switches

### 2.1 p53

p53 coordinates genome-protective programs encompassing DNA damage repair, cell-cycle arrest, senescence, and apoptosis (8). TP53 represents one of the most frequently altered genes across cancers; missense, nonsense, and frameshift mutations can abolish transcriptional activity or confer dominant-negative and gain-of-function effects (9). Importantly, even when TP53 remains wild-type, p53 function is often suppressed in tumors. Oncogenic Ras activates PI3K/Akt, which stimulates the E3 ligase MDM2 to ubiquitinate and degrade p53, while MAPK/ERK signaling attenuates p53 transcriptional output (6). Ras-driven redox remodeling further elevates apoptotic thresholds: enhanced reactive oxygen species (ROS) generation elicits antioxidant responses that blunt p53-mediated death programs and favor survival under stress (10). In parallel, epigenetic mechanisms—histone acetylation/methylation dynamics and DNA methylation at p53 target promoters—can silence apoptotic effectors downstream of p53 (11). These multilayered constraints provide a mechanistic rationale for the limited efficacy of p53 reactivators such as APR-246 when deployed in isolation, before upstream Ras/TME repression is relieved (12). In a sequential paradigm, restoring p53 becomes effective only after dismantling microenvironmental buffering and inhibiting Ras-mediated suppression.

### 2.2 Ras

Ras GTPases integrate signals from receptor tyrosine kinases to drive proliferation, survival, and metabolic remodeling (13). Oncogenic mutations (e.g., KRAS G12C) lock the protein in an active, GTP-bound state, constitutively engaging PI3K/Akt and RAF/MEK/ERK cascades and thereby promoting angiogenesis, glycolysis, and resistance to apoptosis (14). Ras intersects with the p53 axis through several routes: activation of MDM2-mediated degradation, ERK-dependent repression of p53 transcriptional programs, redox adaptation that buffers p53-induced oxidative stress, and chromatin-level silencing of p53-responsive loci (6, 10, 11). Early attempts to inhibit Ras through farnesyltransferase blockade failed because KRAS and NRAS can undergo alternative prenylation, preserving membrane localization and signaling. By contrast, covalent inhibitors of KRAS G12C (e.g., sotorasib, adagrasib) have produced meaningful clinical responses in non–small cell lung cancer, validating the tractability of direct Ras inhibition and providing a critical lever for sequential strategies (14, 15).

### 2.3 HIF-1α

HIF-1α is a master regulator of the hypoxic response. Under normoxia, prolyl hydroxylases mark HIF-1α for von Hippel–Lindau (VHL)-mediated proteasomal degradation. Hypoxia inhibits hydroxylation, stabilizing HIF-1α and enabling transcription of angiogenic (e.g., VEGF) and glycolytic genes that sustain survival under low oxygen (3). Pharmacologic targeting of oxygen-sensing pathways has been clinically validated by HIF-2α inhibition with belzutifan in VHL-associated tumors (16, 17). Nevertheless, broader deployment of HIF-axis inhibitors in common solid tumors will likely require integration with complementary agents that relieve redundant survival circuits or sensitize tumors to apoptosis (**Table 1**).

**Table 1:** Selected targets, representative inhibitors, and clinical experience.

Target	Representative inhibitor(s)	Clinical experience/phase [ref]	Summary outcome
HIF-1α	PX-478	Phase I monotherapy (17)	Preclinical efficacy; clinical use limited by toxicity
HIF-2α (VHL)	Belzutifan	Approved in VHL-associated tumors (16)	Durable responses in VHL disease; validates oxygen-sensing axis
NF-κB (indirect)	Bortezomib	Multiple Phase II/III in solid tumors (18)	Efficacy in hematologic cancers; limited benefit in most solid tumors
KRAS G12C	Sotorasib	Phase II in NSCLC (14)	Objective responses; validates direct Ras inhibition
KRAS G12C	Adagrasib	Phase II in NSCLC (15)	Durable disease control; supports Phase II sequencing
Mutant p53 reactivation	APR-246 (eprenetapopt)	Multiple Phase II/III (12)	Limited benefit when upstream repression persists
MDM2–p53 interaction	RG7112	Phase I (24)	Partial responses and p53 pathway activation

### 2.4 BNIP3

BNIP3 illustrates the context dependence of dual-function proteins. While it can induce mitochondrial outer membrane permeabilization and apoptosis, in hypoxic tumors BNIP3 frequently drives mitophagy, removing dysfunctional mitochondria, limiting ROS accumulation, and thereby promoting survival (5). Therapeutic interventions must therefore tune BNIP3 activity toward pro-apoptotic outputs—either by inhibiting its mitophagic function during early sequencing or by combining it with agents that lower the survival advantage conferred by mitophagy.

### 2.5 NF-κB

NF-κB governs inflammation and innate immunity but is persistently activated in many cancers by TME-derived cytokines, leading to transcription of survival, angiogenic, and immunosuppressive programs (4). Canonical signaling proceeds through IKK-mediated phosphorylation of IκB, p65 nuclear translocation, and activation of target genes that can directly antagonize p53 functions. Clinical experience demonstrates context constraints: proteasome inhibitors such as bortezomib reduce NF-κB activity and are effective in several hematologic malignancies, yet they have generally not reproduced this benefit across solid tumors, reflecting pathway redundancy and microenvironmental buffering (18).

## 3 Sequential therapeutic strategy

A stepwise strategy can exploit the conditional nature of dual-function proteins. Phase I focuses on dismantling TME-driven adaptations by inhibiting hypoxia responses (e.g., HIF-1α/2α), tempering inflammatory signaling (NF-κB), and preventing survival-promoting mitophagy (BNIP3) (3–5). This reduces angiogenesis, lowers glycolytic buffering, and weakens survival circuits that would otherwise absorb pro-apoptotic stimuli. Phase II inhibits Ras, thereby relieving p53 repression via MDM2/ERK pathways and mitigating redox and epigenetic barriers (6, 10, 11, 13–15). Direct KRAS G12C inhibitors provide a clear entry point; in non-G12C settings, upstream RTK or downstream MEK/ERK and PI3K/Akt nodes may be targeted based on tumor dependencies. Phase III restores p53 activity—either by stabilizing wild-type p53 through MDM2 antagonism or by pharmacologically reactivating mutant p53—once upstream suppression has been alleviated (12, 24). Biomarker-guided decision points are essential: hypoxia imaging, cytokine profiles, Ras mutation status, and p53 integrity collectively inform where to begin and how to progress. Within this framework, premature activation of p53 is avoided, as it risks futility when Ras and the TME remain intact.

## 4 Lessons from past therapies

Past experiences underscore the importance of sequencing. APR-246 sought to reactivate mutant p53, but durable benefit was limited—consistent with a model in which p53 remains functionally constrained by Ras-driven and TME-mediated repression when these are not addressed first (12). Bortezomib effectively suppresses NF-κB in hematologic malignancies yet has delivered modest outcomes in most solid tumors, illustrating context dependency and pathway redundancy (18). PX-478, an HIF-1α inhibitor, produced strong preclinical activity but encountered dose-limiting toxicities clinically, limiting its standalone impact (17). Conversely, belzutifan’s success in VHL-associated tumors validates the oxygen-sensing axis as a drug target, while also highlighting that efficacy can be disease-context specific (16). Most notably, covalent KRAS G12C inhibitors achieved meaningful responses in lung cancer, conclusively demonstrating that direct Ras targeting is possible in patients and should be prioritized as the second phase of a sequential strategy where applicable (14, 15) (see **Table 1**).

## 5 Personalized application and biomarkers

Personalization is inherent to sequencing. Hypoxia-dominant tumors may warrant HIF-axis inhibition as Phase I, guided by imaging with radiotracers such as fluoromisonidazole (FMISO) positron emission tomography (19). Inflammation-driven phenotypes, evident from cytokine panels or TME transcriptional profiles, suggest prioritizing NF-κB blockade (20). Ras mutation status dictates feasibility of direct Ras inhibitors; in wild-type Ras tumors with amplified upstream signaling, RTK or pathway-node inhibitors may serve as surrogates (13–15). The status of p53—wild-type but suppressed versus mutant—can guide the use of MDM2 antagonists or reactivators, respectively, once upstream constraints are relieved (12, 24). Liquid biopsy approaches enable longitudinal monitoring of mutation dynamics and clonal selection during sequencing, informing when to switch phases or de-escalate therapy (21). Autophagy and mitophagy markers provide additional signals regarding BNIP3’s role and the degree of mitochondrial stress (5).

## 6 Challenges and TME co-evolution

Several challenges accompany sequential therapy. Tumor heterogeneity generates spatially distinct niches with variable hypoxia, inflammation, Ras pathway activity, and p53 status, complicating uniform biomarker thresholds (22). Combining multiple targeted agents sequentially (and sometimes in overlap windows) increases the risk of cumulative or synergistic toxicities; careful scheduling, dose optimization, and early safety stopping rules are essential (23). Most critically, resistance evolves dynamically. Therapy imposes selective pressures that reshape the TME—reprogramming fibroblasts, endothelial cells, and immune infiltrates—and foster expansion of resistant subclones with alternative survival strategies (7, 22). Adaptive clinical trial designs with predefined biomarker-driven transitions between phases, coupled with serial imaging and liquid biopsies, can operationalize the sequencing concept while minimizing exposure to ineffective regimens (23).

## 7 Conclusion

Dual-function proteins integrate context-dependent survival and death signals. Their rewiring by oncogenic mutations and the TME underlies the persistence of apoptosis-resistant cancer. Historical failures of single-node interventions can be reinterpreted through the lens of persistent Ras-mediated repression of p53 and microenvironmental buffering. A sequential framework—first dismantling TME adaptations, then inhibiting Ras to relieve p53 repression, and finally restoring p53—offers a biologically rational and testable path to restore apoptotic competence. This concept does not solve all resistance mechanisms but provides a clear direction for preclinical development and clinical trials.
